# A comparison of the risk prediction models PERSARC and Sarculator in patients with localized soft tissue sarcoma of the extremities and trunk wall

**DOI:** 10.1016/j.esmoop.2025.105517

**Published:** 2025-07-24

**Authors:** M.R. Kobbeltvedt, I. Lobmaier, M. Spreafico, D. Callegaro, R. Miceli, F. Kizilaslan, D. Swanson, I. Hompland, S. Pasquali, M. Fiocco, M.A.J. van de Sande, A. Gronchi, K. Boye

**Affiliations:** 1 Department of Oncology, Oslo University Hospital, Oslo, Norway; 2 Faculty of Medicine, University of Oslo, Oslo, Norway; 3 Department of Pathology, Oslo University Hospital, Oslo, Norway; 4 Mathematical Institute, Leiden University, Leiden, The Netherlands; 5 Department of Biomedical Data Sciences, Leiden University Medical Centre, Leiden, The Netherlands; 6 Department of Surgery, Fondazione IRCCS Istituto Nazionale Dei Tumori, Milan, Italy; 7 Department of Biostatistics for Clinical Research, Fondazione IRCCS Istituto Nazionale Dei Tumori, Milan, Italy; 8 Oslo Centre for Biostatistics and Epidemiology, Department of Biostatistics, University of Oslo, Oslo, Norway; 9 Department of Biostatistics, University of Texas MD Anderson Cancer Center, Houston, USA; 10 Molecular Pharmacology, Department of Experimental Oncology, Fondazione IRCCS Istituto Nazionale dei Tumori, Milan, Italy; 11 Trial and Data Center, Princess Máxima Center for Pediatric Oncology, Utrecht, The Netherlands; 12 Department of Orthopaedic Oncology, Leiden University Medical Centre, Leiden, The Netherlands

**Keywords:** soft tissue sarcoma, PERSARC, Sarculator, risk models, neoadjuvant/adjuvant chemotherapy

## Abstract

**Background:**

Different risk classification criteria are used to select patients with localized soft tissue sarcoma of the extremities and trunk wall for neoadjuvant/adjuvant chemotherapy. The two most frequently used risk classification methods are PERSARC and Sarculator prediction models. The main aim was to evaluate and compare these two methods.

**Materials and methods:**

The study cohort consisted of 664 patients treated at Oslo University Hospital from 1998 to 2017. Predicted probabilities of distant metastasis (DM) and overall survival (OS) were calculated, and risk classification was carried out according to previously defined thresholds. Hazard ratios were estimated using Cox proportional hazards regression models. Interaction between neoadjuvant/adjuvant chemotherapy and risk groups was included in the models to investigate the effect of chemotherapy according to risk group.

**Results:**

A high degree of correlation was found between PERSARC and Sarculator in predicted 5-year probability of DM and 5-year OS. A total of 215 of 664 (32%) and 221 of 569 (39%) patients were classified as high-risk according to Sarculator and PERSARC, respectively, with agreement found in 511 of 569 patients (90%). Patients classified as high-risk by only one method had similar disease-free survival and OS as patients who were high-risk using both methods. Based on this, patients classified as high-risk by at least one method were grouped as ‘combined high-risk’ and compared with previously established risk classification criteria. Neoadjuvant/adjuvant chemotherapy was associated with improved OS and disease-free survival in all high-risk groups.

**Conclusions:**

A high degree of agreement between PERSARC and Sarculator predictions was observed. Patients classified as high-risk by only one method had similar outcomes to those who were high-risk using both. Chemotherapy was associated with improved outcome in the PERSARC, Sarculator, and combined high-risk group. Patients classified as high-risk by one of these methods could be considered for neoadjuvant/adjuvant chemotherapy.

## Highlights

• The risk classification models PERSARC and Sarculator showed a high degree of agreement.
• Patients classified as high-risk by only one method had similar outcome as patients who were high-risk using both methods.
• Perioperative chemotherapy was associated with improved outcome in all high-risk groups.
• We propose that patients classified as high-risk by at least one method should be defined as high-risk.

## Introduction

Around 25% of patients with localized soft tissue sarcoma (STS) of the extremities and trunk wall develop distant metastasis (DM) following curatively intended local treatment.^1^ Precise prediction of prognosis of individual patients is challenging due to large differences in biological behavior, >50 different histological types, and a relatively low incidence. Identification of patients at high risk of disease recurrence is crucial, as these individuals may benefit from neoadjuvant/adjuvant chemotherapy to prevent disease relapse.^2^^,^^3^ However, meta-analyses of randomized trials have yielded conflicting evidence regarding the overall survival (OS) benefit.^4^ Furthermore, chemotherapy is associated with potentially harmful side-effects and patients with a low risk of recurrence should be spared a toxic treatment with minimal or no benefit.

Several risk classification systems are used to select patients for chemotherapy. The American Joint Committee on Cancer (AJCC) staging system is based on TNM (tumor–node–metastasis) stage and histological grade according to the FNCLCC (Fédération Nationale des Centers De Lutte Contre Le Cancer) grading system.^5^^,^^6^ In some randomized trials, high-grade, deep-seated tumors >5 cm have been considered high-risk.^7^, ^8^, ^9^ In phase II trials conducted by the Scandinavian Sarcoma Group (SSG), high-risk patients were selected based on tumor size, vascular invasion, tumor necrosis, and infiltrating growth pattern.^10^^,^^11^ The main limitations of the aforementioned systems are that histological type is not considered and that an individualized estimate of outcome is not provided. Relying solely on size and grade criteria to select patients for chemotherapy trials results in a highly imprecise risk prediction, as it overlooks the significant heterogeneity in the individual patient risk, which is influenced by factors beyond these two parameters.^12^

Prognostic nomograms enable individualized predictions of prognosis and are increasingly used by clinicians and patients to support clinical decision making. Since the first nomogram was published in 2002 by Kattan and colleagues,^13^ several risk prediction models have been developed. Today, two of the most used models are PERSARC and Sarculator.^14^^,^^15^ The variables included in Sarculator are age, tumor size, FNCLCC grade, and histological type. PERSARC includes age, sex, tumor size, histological type, tumor depth, and FNCLCC grade (only 2 and 3). Patients with a 10-year OS probability of ≤60% according to Sarculator are classified as high-risk, based on retrospective analyses of randomized trials that demonstrated a benefit of neoadjuvant/adjuvant chemotherapy in this subgroup.^3^^,^^16^^,^^17^ Similarly, PERSARC defines high risk as a probability of 5-year OS ≤66%, based on data from multiple sarcoma reference centers.^2^ The two models have been validated externally,^18^^,^^19^ but they have never been compared except in one study in malignant peripheral nerve sheath tumors.^20^

In this study, the primary aim was to evaluate the two risk prediction models PERSARC and Sarculator in a population-based cohort of patients with localized STS of the extremities and trunk wall and to compare risk classification based on PERSARC and Sarculator. Furthermore, we compared the two models with other risk classification systems used to select patients for neoadjuvant/adjuvant chemotherapy.

## Materials and methods

### Patients

Adult patients (≥18 years) treated for primary, localized soft tissue sarcoma of the extremities or trunk wall between 1998 and 2017 at Oslo University Hospital (OUH) were identified from a prospectively maintained institutional database, as previously described.^21^ The prospectively collected data in the database were supplemented by retrospective review of medical records. The study was approved by the Regional Committee for Medical Research Ethics South East Norway (approval no. 661025). A waiver of written informed consent was granted. All patients were provided the required consent information.

### Diagnosis, treatment, and follow-up

Malignancy grading was carried out according to the FNCLCC grading system.^5^ The 2020 World Health Organization classification was used for histopathological diagnosis.^22^ All specimens were reviewed by an expert sarcoma pathologist as part of routine diagnostics. Additionally, all pathology reports were reviewed for the current study, and in 296 cases, the histological specimen was reviewed by a reference sarcoma pathologist (IL). Local treatment, neoadjuvant/adjuvant chemotherapy, and follow-up protocols have been described previously.^21^

### Risk prediction models

Data from each patient were entered into the two risk prediction models PERSARC and Sarculator. We estimated the OS and probability of DM at 5 years after surgery using PERSARC and OS and probability of DM at 5 and 10 years using Sarculator. Patients were classified as high-risk by PERSARC if the 5-year predicted OS (prOS) was ≤66% and as high-risk by Sarculator if the 10-year prOS was ≤60%.^2^^,^^16^ Additionally, patients were also categorized into different high-risk categories based on the following alternative criteria: (i) deep-seated tumor, size ≥5 cm and FNCLCC grade 3; (ii) deep-seated tumor, size ≥5 cm and FNCLCC grade 2 or 3; (iii) AJCC stage III; and (iv) the criteria used in the SSG XX study,^11^ i.e. FNCLCC grade II or III and vascular invasion and/or at least two of the following criteria: size ≥8.0 cm, infiltrative growth pattern, and tumor necrosis. Note that patients classified as high-risk according to the alternative criteria could differ from those classified as high-risk by PERSARC and Sarculator, since the underlying definitions are distinct. For each of the aforementioned risk models, we calculated median PERSARC 5-year prOS and Sarculator 5- and 10-year prOS with interquartile range.

### Statistical methods

OS time was calculated from the date of surgery to the date of death from any cause. Disease-free survival (DFS) time was calculated from the date of surgery to the date of DM, local recurrence, or death from any cause. For both outcomes, patients who had not experienced an event were censored at the date of last follow-up. Survival probability was estimated using the Kaplan–Meier method. Median follow-up time was calculated using the reverse Kaplan–Meier method.^23^ Estimated Kaplan–Meier curves were compared using the log-rank test. Hazard ratios (HRs) were derived from Cox proportional hazards regression models. Correlation between 5-year OS or 5-year probability of DM predicted by PERSARC and Sarculator was calculated using the Pearson correlation coefficient (r). Seven separate Cox regression models were constructed, each including two binary variables: one defining the risk group (low- versus high-risk) based on each of the seven classification criteria, and another indicating whether neoadjuvant/adjuvant chemotherapy was administered (yes/no). Additionally, the interaction between the two binary variables was included to allow for the estimation of the effect of neoadjuvant/adjuvant chemotherapy within the high-risk group defined by each classification system. Statistical analyses were carried out in the Statistical Package for the Social Sciences (SPSS) for Windows version 29.0 (Armonk, NY) and in the R software environment.^24^

## Results

### Study population

The study population was based on a previously published cohort of patients treated at the Oslo University Hospital from 1998 to 2017.^21^ One hundred and twenty-six patients were excluded from the present study due to missing grade (*n* = 72), primary surgery outside a sarcoma center without secondary surgery at OUH (*n* = 29), missing tumor size (*n* = 24), and cutaneous tumor location (*n* = 1). After pathology review, another 16 patients were excluded due to the revised diagnosis [BCL-6 interacting corepressor-associated sarcoma, angiomatoid fibrous histiocytoma, atypical lipomatous tumor, Epstein–Barr virus-associated leiomyomatosis, perivascular epithelioid cell tumor (PEComa), solitary fibrous tumor, and uncertain diagnosis]. Thus, the study cohort consisted of 664 patients. A subgroup of 569 patients met the requirements for both PERSARC and Sarculator and was used for further comparison between the two models. The characteristics of the total cohort and the subgroup are summarized in Table 1. The most frequent histological subtypes were myxofibrosarcoma (*n* = 184), undifferentiated pleomorphic sarcoma (UPS; *n* = 129), and leiomyosarcoma (*n* = 92). Ninety-five patients (14%) had FNCLCC grade I tumors, while 569 patients (86%) had grade II or III tumors.

**Table 1:** Demographic, clinical, and pathological characteristics of the patient cohort

	Total cohort *n* = 664 (%)	Subgroup for comparisons *n* = 569 (%)
Age at surgery, median (range)	60 (18-94)	63 (18-94)
Sex		
Female	306 (46.1)	265 (46.6)
Male	358 (53.9)	304 (53.4)
Primary tumor location		
Lower extremity	391 (58.9)	339 (59.6)
Upper extremity	121 (18.2)	94 (16.5)
Trunk wall	152 (22.9)	136 (23.9)
Histological type		
Myxofibrosarcoma	184 (27.7)	167 (29.3)
UPS	129 (19.4)	129 (22.7)
Leiomyosarcoma	92 (13.9)	73 (12.8)
Synovial sarcoma	49 (7.4)	49 (8.6)
Liposarcoma		
Pleomorphic	30 (4.5)	30 (5.3)
Dedifferentiated	15 (2.2)	15 (2.6)
Not specified	1 (0.2)	1 (0.2)
Myxoid liposarcoma	37 (5.6)	7 (1.2)
MPNST	24 (3.6)	21 (3.7)
Extraskeletal myxoid chondrosarcoma	15 (2.3)	10 (1.8)
Low-grade fibromyxoid sarcoma	13 (2.0)	
Angiosarcoma	10 (1.5)	10 (1.8)
Not classified	32 (4.8)	27 (4.7)
Other^a^	33 (5.0)	30 (5.3)
Tumor size (cm), median (range)	8.3 (0.6-40)	7.5 (0.6-40)
Tumor depth		
Superficial	205 (30.9)	171 (30.1)
Deep	459 (69.1)	398 (69.9)
Malignancy grade		
FNCLCC grade 1	95 (14.3)	
FNCLCC grade 2	356 (53.6)	356 (62.6)
FNCLCC grade 3	213 (32.1)	213 (37.4)
Primary surgery OUH		
Yes	543 (81.8)	464 (81.6)
No	121 (18.2)	105 (18.5)
Surgical margin		
R0	585 (88.1)	500 (87.9)
R1	79 (11.9)	69 (12.1)
Neoadjuvant/adjuvant chemotherapy		
Yes	150 (22.6)	147 (25.8)
No	514 (77.4)	422 (74.2)
Neoadjuvant/adjuvant radiotherapy		
Yes	378 (56.9)	355 (62.4)
No	286 (43.1)	214 (37.6)

FNCLCC, Fédération Nationale des Centers de Lutte Contre Le Cancer; MPNST, malignant peripheral nerve sheath tumor; OUH, Oslo University Hospital; R0, complete resection with microscopically negative margins; R1, complete resection with microscopically positive margins; UPS, undifferentiated pleomorphic sarcoma.

a Other subtypes included extraskeletal osteosarcoma (9), epithelioid sarcoma (6), fibrosarcoma (3), sclerosing epithelioid fibrosarcoma (3), pleomorphic dermal sarcoma (2), clear cell sarcoma (2), pleomorphic rhabdomyosarcoma (3), sclerosing rhabdomyosarcoma (1), granular cell tumor (1), epithelioid hemangioendothelioma (1), myxoinflammatory fibroblastic sarcoma (1), and alveolar soft part sarcoma (1). In the comparison subgroup, three patients with epithelioid hemangioendothelioma, myxoinflammatory fibroblastic sarcoma, and sclerosing epithelioid fibrosarcoma were excluded.

### Treatment

All patients underwent complete resection for primary STS at OUH or were reoperated at OUH after initial surgery. A microscopically negative margin (R0) was achieved in 88%. Pre- or post-operative radiotherapy was administered to 378 (57%) patients. One hundred and fifty patients (23%) received chemotherapy, of whom 14 were treated with neoadjuvant chemotherapy, 128 with adjuvant chemotherapy, and 8 with both neoadjuvant and adjuvant chemotherapy. One hundred and thirty-seven patients (91%) received a regimen with an anthracycline and ifosfamide.

### PERSARC

Median 5-year prOS calculated with PERSARC was 72.5% (range 1.3%-96.3%) (Supplementary Figure S1, available at https://doi.org/10.1016/j.esmoop.2025.105517). Among 569 patients with FNCLCC grade II or III tumors for whom predicted survival could be estimated using PERSARC, 221 (39%) patients were identified as high-risk and 348 (61%) patients as low-risk. The median 5-year prOS in the high-risk group was 50.5% (range 1.3%-65.9%) and in the low-risk group was 82.4% (range 66.1%-96.3%). Fifty (23%) high-risk patients and 97 (28%) low-risk patients received neoadjuvant/adjuvant chemotherapy.

### Sarculator

Median 5- and 10-year prOS with Sarculator was 80.7% (range 3.2%-98.6%) and 72.0% (range 0.5%-97.9%), respectively. In the subcohort of 569 patients available for assessment with PERSARC, the median 5-year prOS was 77.5% (range 3.2%-97.3%; Supplementary Figure S1, available at https://doi.org/10.1016/j.esmoop.2025.105517), and 215 (38%) patients were classified as high-risk and 354 (62%) as low-risk. The median 5-year prOS was 58.9% (range 3.3%-71.6%) for the high-risk group and 84.7% (range 71.6%-97.3%) in the low-risk group. In the high-risk group, 58 (27%) patients received neoadjuvant/adjuvant chemotherapy, while 92 (21%) received neoadjuvant/adjuvant chemotherapy in the low-risk group.

### Comparison of PERSARC and Sarculator

**Figure 1:**
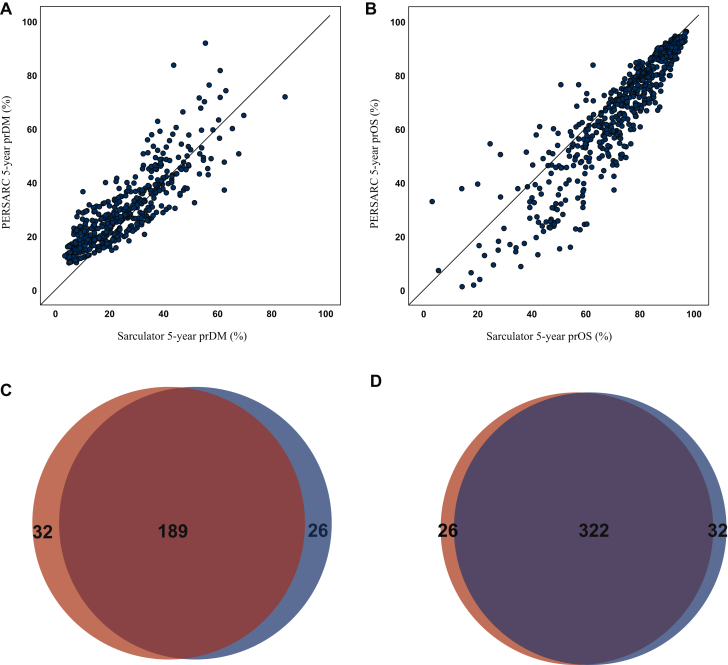
**Comparison between PERSARC and Sarculator.** (A, B) Scatter plot showing the correlation between the 5-year probability of distant metastasis (prDM; A) and 5-year predicted overall survival (prOS; B) using PERSARC and Sarculator as indicated. (C, D) Venn diagrams illustrating the relationship between patients classified as high-risk (C) and low-risk (D) by PERSARC (red) and Sarculator (blue).

We found a high correlation between PERSARC and Sarculator for predicted 5-year probability of DM [Figure 1A; r = 0.849; 95% confidence interval (CI) 0.825-0.871] and for predicted 5-year OS (Figure 1B; r = 0.908; 95% CI 0.892-0.921). One hundred and eighty-nine patients (33%) were classified as high-risk and 322 (57%) as low-risk by both models (Figure 1C and D). Thus, the methods were in agreement in 511 patients (90%). Fifty-eight patients were classified as high-risk by only one method, of whom 32 were PERSARC high-risk and 26 Sarculator high-risk. The characteristics of this patient group are summarized in Supplementary Table S1, available at https://doi.org/10.1016/j.esmoop.2025.105517.

In the 32 patients classified as high-risk by PERSARC and low-risk by Sarculator, UPS (*n* = 14) and myxofibrosarcoma (*n* = 9) were the most frequent subtypes. There was no particularly notable histological types in the 26 patients classified as high-risk by Sarculator and low-risk by PERSARC. In the PERSARC high-risk Sarculator low-risk group (*n* = 32), 19% received chemotherapy and 28% radiotherapy, while 54% was treated with chemotherapy and 89% with radiotherapy in the Sarculator high-risk PERSARC low-risk group (*n* = 26).

### Patient outcome

**Figure 2:**
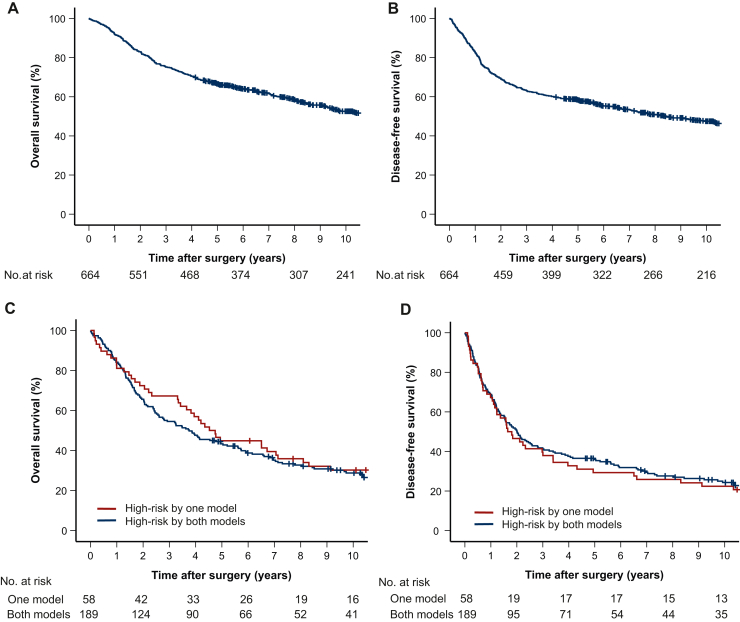
**Patient outcome.** Kaplan–Meier curves showing overall survival (A) and disease-free survival (B) for the total cohort (*n* = 664), and overall survival (C) and disease-free survival (D) stratified based on whether patients were classified as high-risk by only one risk model (orange) or both (blue).

After a median follow-up for OS of 155 months, 349 (52%) patients had died. Of these 349 patients, 202 (58%) had been diagnosed with local recurrence or DM. Median OS was 88 months, and estimated 5- and 10-year OS was 67% and 53%, respectively (Figure 2A). The median follow-up for DFS was 154 months. Local recurrence was diagnosed in 39 (6%) patients, DM in 165 (25%) patients, and combined local recurrence and DM in 30 (5%) patients. Lung (77%) was the most frequent metastatic site. Median DFS was 69 months (Figure 2B). There was no difference in DFS (HR 1.10, 95% CI 0.86-1.39) or OS (HR 1.04, 95% CI 0.81-1.34) between patients with primary tumors of the trunk wall and extremities. The outcome data of the total cohort and the high-risk groups are summarized in Supplementary Table S2, available at https://doi.org/10.1016/j.esmoop.2025.105517. OS and DFS for PERSARC and Sarculator low- and high-risk are shown in Supplementary Figure S2, available at https://doi.org/10.1016/j.esmoop.2025.105517. Patients classified as high-risk by only one method (*n* = 58; Supplementary Table S1, available at https://doi.org/10.1016/j.esmoop.2025.105517) had similar OS as patients who were high-risk using both methods (Figure 2C; HR 1.11, 95% CI 0.79-1.55). DFS was also comparable between the two groups (Figure 2D; HR 0.94, 95% CI 0.68-1.30). Based on this observation, we generated a combined high-risk group where patients classified as high-risk by at least one method were defined as high-risk. The combined high-risk group consisted of 247 patients (Supplementary Table S3, available at https://doi.org/10.1016/j.esmoop.2025.105517). Median OS in this group was 47 months (range 0.1-288 months), 5-year OS was 44%, and 10-year OS was 29%. Median DFS was 23 months, and 5-year and 10-year DFS was 34% and 24%, respectively (Supplementary Figure S3, available at https://doi.org/10.1016/j.esmoop.2025.105517).

### Co*mparison with other risk criteria*

The new proposed classification of high-risk based on the combination of PERSARC and Sarculator (‘combined high-risk’) was compared with previously used risk criteria.^6^^,^^9^^,^^11^^,^^25^ In our total cohort of 664 patients, 154 (23%) were grade 3, deep-seated, and ≥5 cm, whereas 332 (50%) were deep-seated, ≥5 cm, and FNCLCC grade 2-3 (Table 2). Furthermore, 402 (61%) patients had AJCC stage III tumors. According to the criteria used in the SSG XX study, 376 of 565 (57%) assessable patients were classified as high-risk. Only ∼60% of patients with deep-seated, high-grade tumors ≥5 cm, AJCC stage III, and SSG XX high-risk were high-risk according to the combined PERSARC/Sarculator model (Supplementary Figure S4, available at https://doi.org/10.1016/j.esmoop.2025.105517). On the other hand, >85% of those classified as high-risk by the combined model were also high-risk using the other criteria, except for deep-seated, grade 3 tumors ≥5 cm (Table 2). Patients with deep-seated, high-grade tumors ≥5 cm, AJCC stage III tumors, and SSG XX high-risk had better predicted OS based on PERSARC and Sarculator compared with the other high-risk groups (Table 2 and Supplementary Figure S5, available at https://doi.org/10.1016/j.esmoop.2025.105517).

**Table 2:** Comparison of risk models

	Low-risk combined	High-risk combined	PERSARC 5-year prOS	Sarculator 5-year prOS	Sarculator 10-year prOS	Total
Deep-seated, G3, ≥5 cm						
No	390 (76.5)	120 (23.5)	79.5 (66.3-88.7)	85.3 (74.4-92.4)	78.5 (63.6-88.7)	510
Yes	27 (17.5)	127 (82.5)	53.3 (34.4-64.2)	66.2 (47.4-71.8)	46.1 (61.9-60.3)	154
Deep-seated, G2-3, ≥5 cm						
No	297 (89.0)	35 (11.0)	86.6 (75.3-90.6)	90.7 (83.5-94.2)	88.2 (82.5-92.6)	332
Yes	120 (36.1)	212 (63.9)	61.5 (48.3-74.4)	69.9 (57.0-78.5)	59.1 (43.3-70.6)	332
AJCC stage						
Stage I-II	254 (96.9)	8 (3.1)	89.5 (80.8-91.6)	92.1 (88.2-95.1)	88.2 (85.2-92.6)	262
Stage III	163 (40.5)	239 (59.5)	64.0 (49.2-76.5)	70.1 (57.8-79.6)	59.1 (43.3-70.6)	402
SSG XX criteria						
Negative	182 (96.3)	7 (3.7)	87.4 (80.0-91.1)	92.1 (87.1-95.3)	88.2 (81.0-92.9)	189
Positive	151 (40.2)	225 (59.8)	63.0 (48.3-76.0)	70.4 (57.1-79.8)	58.5 (42.5-70.9)	376
Not determined	84 (84.8)	15 (15.2)				99
Total	417 (62.8)	247 (37.2)				664

Data are numbers of patients (%). Percentages are calculated by rows. Predicted overall survival (prOS) is presented as median with interquartile range.

AJCC, American Joint Committee on Cancer; G, Fédération Nationale des Centers De Lutte Contre Le Cancer grade; SSG, Scandinavian Sarcoma Group.

### Neoadjuvant/adjuvant chemotherapy

In the combined high-risk group, neoadjuvant/adjuvant chemotherapy was associated with improved OS and DFS (Table 3; model 1). Comparable results were obtained in the PERSARC and Sarculator high-risk groups, and for patients with deep-seated, grade 3 tumors ≥5 cm (models 2-4, respectively). For patients with deep-seated, high-grade tumors ≥5 cm (model 5), AJCC stage III tumors (model 6) and SSG XX high-risk tumors (model 7), a benefit of neoadjuvant/adjuvant chemotherapy was also observed.

**Table 3:** Estimated hazard ratios and 95% confidence intervals for overall survival and disease-free survival

Model	Classification system	HR_OS_ (95% CI)	HR_DFS_ (95% CI)
1	Combined high-risk	0.40 (0.28-0.57)	0.44 (0.31-0.62)
2	PERSARC high-risk	0.36 (0.24-0.54)	0.41 (0.28-0.61)
3	Sarculator high-risk	0.38 (0.26-0.56)	0.43 (0.19-0.62)
4	Deep-seated, G3, ≥5 cm	0.33 (0.21-0.50)	0.38 (0.25-0.57)
5	Deep-seated, G2-3, ≥5 cm	0.52 (0.39-0.69)	0.55 (0.42-0.72)
6	AJCC stage III	0.55 (0.42-0.73)	0.59 (0.45-0.76)
7	SSG XX criteria	0.49 (0.37-0.65)	0.54 (0.41-0.71)

Data are presented as hazard ratios with 95% confidence intervals.

AJCC, American Joint Committee on Cancer; CI, confidence interval; DFS, disease-free survival; G, Fédération Nationale des Centers De Lutte Contre Le Cancer grade; HR, hazard ratio; OS, overall survival; SSG, Scandinavian Sarcoma Group.

## Discussion

In this study, we investigated and compared the two risk models PERSARC and Sarculator in a population-based cohort of patients with STS, and further compared them with previously used risk criteria. We found a high level of agreement between risk classification based on PERSARC and Sarculator predictions. Almost 90% of patients were classified in the same risk group with both models, and predicted OS and the probability of DM were highly correlated. While the two underlying models and the classification of patients into high- or low-risk groups differs to some extent, the overall results are largely overlapping in terms of risk group categorization, even if not identical in point estimates. This is an important finding with potential implications for clinical practice. The two models are widely used and our analysis shows that the choice of risk model has minimal impact on the broader risk classification, underscoring their comparable utility in clinical decision making. Furthermore, we found that patients classified as high-risk by only one of the two models had similar outcome to those classified as high-risk by both models. We created a combined high-risk group based on these findings, which included patients classified as high-risk by at least one model. Previous studies have shown that neoadjuvant/adjuvant chemotherapy is associated with improved outcome in high-risk patients, classified both using PERSARC and Sarculator, but not in the low-risk group.^2^^,^^3^^,^^16^ In our cohort, administration of chemotherapy was also associated with better OS and DFS in PERSARC and Sarculator high-risk patients. The benefit was of similar magnitude in the combined high-risk group as in PERSARC high-risk and Sarculator high-risk. Taken together, patients classified as high-risk by either PERSARC, Sarculator, or both should be considered as high-risk. Our findings also suggest that patients in this combined high-risk group may benefit from neoadjuvant/adjuvant chemotherapy.

Several additional risk classification systems have been used to select high-risk patients for neoadjuvant/adjuvant chemotherapy, and we compared our combined high-risk group with the most commonly used high-risk criteria.^6^^,^^9^^,^^11^^,^^25^ The high-risk groups based on deep-seated, G2-3, ≥5 cm, AJCC stage, and SSG XX were considerably larger than the combined high-risk group (Table 2). Thus, with these criteria a larger number of patients would be offered chemotherapy. They also had a better predicted OS, indicating that patients with a more favorable prognosis and potentially a lower benefit of chemotherapy would be included. On the other hand, deep-seated, G3 tumors ≥5 cm defined a considerably smaller group, where almost half of the combined high-risk patients were not included. There is a risk that this criterion is too stringent, and it also excludes all subcutaneous tumors regardless of their biological characteristics. Based on our results and previous findings,^2^^,^^3^^,^^16^ we suggest that combined high-risk patients should be considered for neoadjuvant/adjuvant chemotherapy.

The dichotomization into low and high risk based on predefined thresholds has obvious limitations. Patients with a predicted OS marginally better than the threshold probably have a similar prognosis as those with a predicted OS marginally worse than the threshold. In clinical decision making, thresholds must always be interpreted wisely and the predicted prognosis for the individual patient should be considered and discussed with the patient. Within the high-risk groups, those at highest risk may also benefit more from systemic treatment compared with those with a more favorable prognosis. Since the thresholds for both PERSARC and Sarculator have been previously defined and validated, we did not aim to explore other cut-offs in this work.

Even though the available models identify patients who could benefit from chemotherapy, there is still a large potential for improvement. In our study, almost half of the patients in the high-risk group who only underwent local therapy did not experience distant recurrence, and these patients obviously do not need additional systemic therapy. On the other hand, approximately half of the high-risk patients who received neoadjuvant/adjuvant chemotherapy developed metastatic disease despite the adjuvant treatment. Future improvements to the risk models may include imaging biomarkers^26^ or molecular biomarkers, such as the gene expression signature CINSARC (complexity index in sarcomas),^27^ genomic complexity,^28^ or proteomic signatures.^29^

Our study has several strengths. We had a sizable patient population, all patients were treated and followed up at the same sarcoma reference center, pathology review was conducted by an expert sarcoma pathologist, and the study cohort was population based as previously described.^21^ Furthermore, during the entire study period patients were followed up at our center with physical examination and radiological imaging (usually chest X-ray) at each visit after end of treatment, providing high-quality data on long-term follow-up. The study also has certain limitations. Some patients had to be excluded due to missing data on malignancy grade and tumor size and certain variables also had missing data for patients included in the cohort, such as for the high-risk criteria in the SSG XX trial. We routinely used chest X-ray for detection of lung metastasis during follow-up and routine chest computed tomography would probably detect metastatic disease earlier. Our study is retrospective, with the well-known inherent limitations of a retrospective design, although patients were prospectively registered in our institutional database. Finally, the study covers a long time period, and diagnostics and treatment may have changed during these years. However, no clear differences in baseline characteristics, outcome, or correlations between PERSARC and Sarculator were found when we compared the former and latter time periods (data not shown).

In conclusion, in this population-based cohort of patients with localized STS of the extremities and trunk wall, we found a high degree of agreement in the high-/low-risk classification between the PERSARC and Sarculator models. Patients classified as high-risk by one model and low-risk by the other had similar outcome as those who were classified as high-risk using both, and we propose that patients classified as high-risk by at least one method should be defined as high-risk. In this combined high-risk group, chemotherapy was associated with improved outcome, suggesting that combined high-risk patients could be considered for neoadjuvant/adjuvant chemotherapy.

## References

[1] Gamboa A.C., Gronchi A., Cardona K. (2020). Soft-tissue sarcoma in adults: an update on the current state of histiotype-specific management in an era of personalized medicine. CA Cancer J Clin.

[2] Acem I., van Houdt W.J., Grunhagen D.J. (2022). The role of perioperative chemotherapy in primary high-grade extremity soft tissue sarcoma: a risk-stratified analysis using PERSARC. Eur J Cancer.

[3] Pasquali S., Palmerini E., Quagliuolo V. (2022). Neoadjuvant chemotherapy in high-risk soft tissue sarcomas: a Sarculator-based risk stratification analysis of the ISG-STS 1001 randomized trial. Cancer.

[4] Goh M.H., Gonzalez M.R., Heiling H.M. (2025). Adjuvant chemotherapy in localized, resectable extremity and truncal soft tissue sarcoma and survival outcomes – a systematic review and meta-analysis of randomized controlled trials. Cancer.

[5] Trojani M., Contesso G., Coindre J.M. (1984). Soft-tissue sarcomas of adults; study of pathological prognostic variables and definition of a histopathological grading system. Int J Cancer.

[6] Amin M.B., Greene F.L., Edge S.B. (2017). The Eighth Edition AJCC Cancer Staging Manual: Continuing to build a bridge from a population-based to a more “personalized” approach to cancer staging. CA Cancer J Clin.

[7] Frustaci S., Gherlinzoni F., De Paoli A. (2001). Adjuvant chemotherapy for adult soft tissue sarcomas of the extremities and girdles: results of the Italian randomized cooperative trial. J Clin Oncol.

[8] Gronchi A., Frustaci S., Mercuri M. (2012). Short, full-dose adjuvant chemotherapy in high-risk adult soft tissue sarcomas: a randomized clinical trial from the Italian Sarcoma Group and the Spanish Sarcoma Group. J Clin Oncol.

[9] Gronchi A., Ferrari S., Quagliuolo V. (2017). Histotype-tailored neoadjuvant chemotherapy versus standard chemotherapy in patients with high-risk soft-tissue sarcomas (ISG-STS 1001): an international, open-label, randomised, controlled, phase 3, multicentre trial. Lancet Oncol.

[10] Jebsen N.L., Bruland O.S., Eriksson M. (2011). Five-year results from a Scandinavian sarcoma group study (SSG XIII) of adjuvant chemotherapy combined with accelerated radiotherapy in high-risk soft tissue sarcoma of extremities and trunk wall. Int J Radiat Oncol Biol Phys.

[11] Sundby Hall K., Bruland Ø.S., Bjerkehagen B. (2018). Adjuvant chemotherapy and postoperative radiotherapy in high-risk soft tissue sarcoma patients defined by biological risk factors-A Scandinavian Sarcoma Group study (SSG XX). Eur J Cancer.

[12] Pasquali S., Colombo C., Pizzamiglio S. (2018). High-risk soft tissue sarcomas treated with perioperative chemotherapy: improving prognostic classification in a randomised clinical trial. Eur J Cancer.

[13] Kattan M.W., Leung D.H., Brennan M.F. (2002). Postoperative nomogram for 12-year sarcoma-specific death. J Clin Oncol.

[14] van Praag V.M., Rueten-Budde A.J., Jeys L.M. (2017). A prediction model for treatment decisions in high-grade extremity soft-tissue sarcomas: personalised sarcoma care (PERSARC). Eur J Cancer.

[15] Callegaro D., Miceli R., Bonvalot S. (2016). Development and external validation of two nomograms to predict overall survival and occurrence of distant metastases in adults after surgical resection of localised soft-tissue sarcomas of the extremities: a retrospective analysis. Lancet Oncol.

[16] Pasquali S., Pizzamiglio S., Touati N. (2019). The impact of chemotherapy on survival of patients with extremity and trunk wall soft tissue sarcoma: revisiting the results of the EORTC-STBSG 62931 randomised trial. Eur J Cancer.

[17] Gronchi A., Palmerini E., Quagliuolo V. (2020). Neoadjuvant chemotherapy in high-risk soft tissue sarcomas: final results of a randomized trial from Italian (ISG), Spanish (GEIS), French (FSG), and Polish (PSG) Sarcoma Groups. J Clin Oncol.

[18] Rueten-Budde A.J., van Praag V.M., van de Sande M.A.J., Fiocco M., PERSARC Study Group (2021). External validation and adaptation of a dynamic prediction model for patients with high-grade extremity soft tissue sarcoma. J Surg Oncol.

[19] Callegaro D., Miceli R., Bonvalot S. (2019). Development and external validation of a dynamic prognostic nomogram for primary extremity soft tissue sarcoma survivors. EClinicalMedicine.

[20] Acem I., Steyerberg E.W., Spreafico M. (2024). Survival after resection of malignant peripheral nerve sheath tumors: introducing and validating a novel type-specific prognostic model. Neurooncol Adv.

[21] Boye K., Lobmaier I., Kobbeltvedt M.R. (2022). Real-world evidence on perioperative chemotherapy in localized soft tissue sarcoma of the extremities and trunk wall; a population-based study. Acta Oncol.

[22] IARC (2020).

[23] Schemper M., Smith T.L. (1996). A note on quantifying follow-up in studies of failure time. Control Clin Trials.

[24] R Core Team (2023).

[25] Gronchi A., Miah A.B., Dei Tos A.P. (2021). Soft tissue and visceral sarcomas: ESMO-EURACAN-GENTURIS Clinical Practice Guidelines for diagnosis, treatment and follow-up. Ann Oncol.

[26] O’Connor J.P., Aboagye E.O., Adams J.E. (2017). Imaging biomarker roadmap for cancer studies. Nat Rev Clin Oncol.

[27] Chibon F., Lagarde P., Salas S. (2010). Validated prediction of clinical outcome in sarcomas and multiple types of cancer on the basis of a gene expression signature related to genome complexity. Nat Med.

[28] Boye K., Gorunova L., Gunawan B. (2023). Genomic complexity as a biomarker to de-escalate adjuvant imatinib treatment in high-risk gastrointestinal stromal tumor. JCO Precis Oncol.

[29] Chadha M., Iadecola S., Jenks A. (2024). Proteomic profiling improves prognostic risk stratification of the Sarculator nomogram in soft tissue sarcomas of the extremities and trunk wall. Cancer Med.

