# Supplementary material for: A comparison of the risk prediction models PERSARC and Sarculator in patients with localized soft tissue sarcoma of the extremities and trunk wall
Source: ESMO Open. 2025 Jul 24;10(8):105517. doi: 10.1016/j.esmoop.2025.105517 (PMC12312030; doi:10.1016/j.esmoop.2025.105517)
Supplement: Supplementary Figures [file mmc1.pptx]

## Slide 1
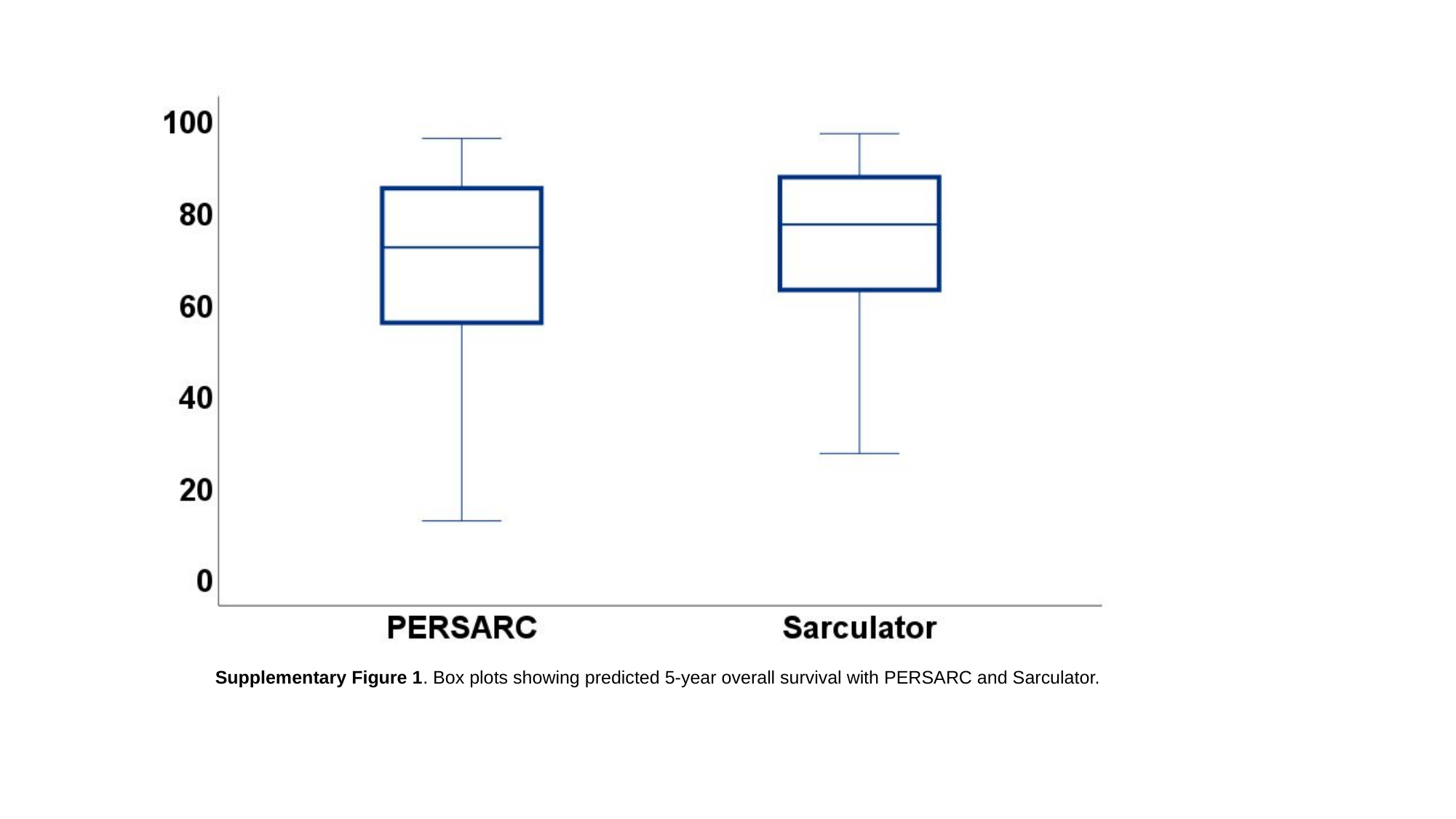

Supplementary Figure 1. Box plots showing predicted 5-year overall survival with PERSARC and Sarculator.

## Slide 2
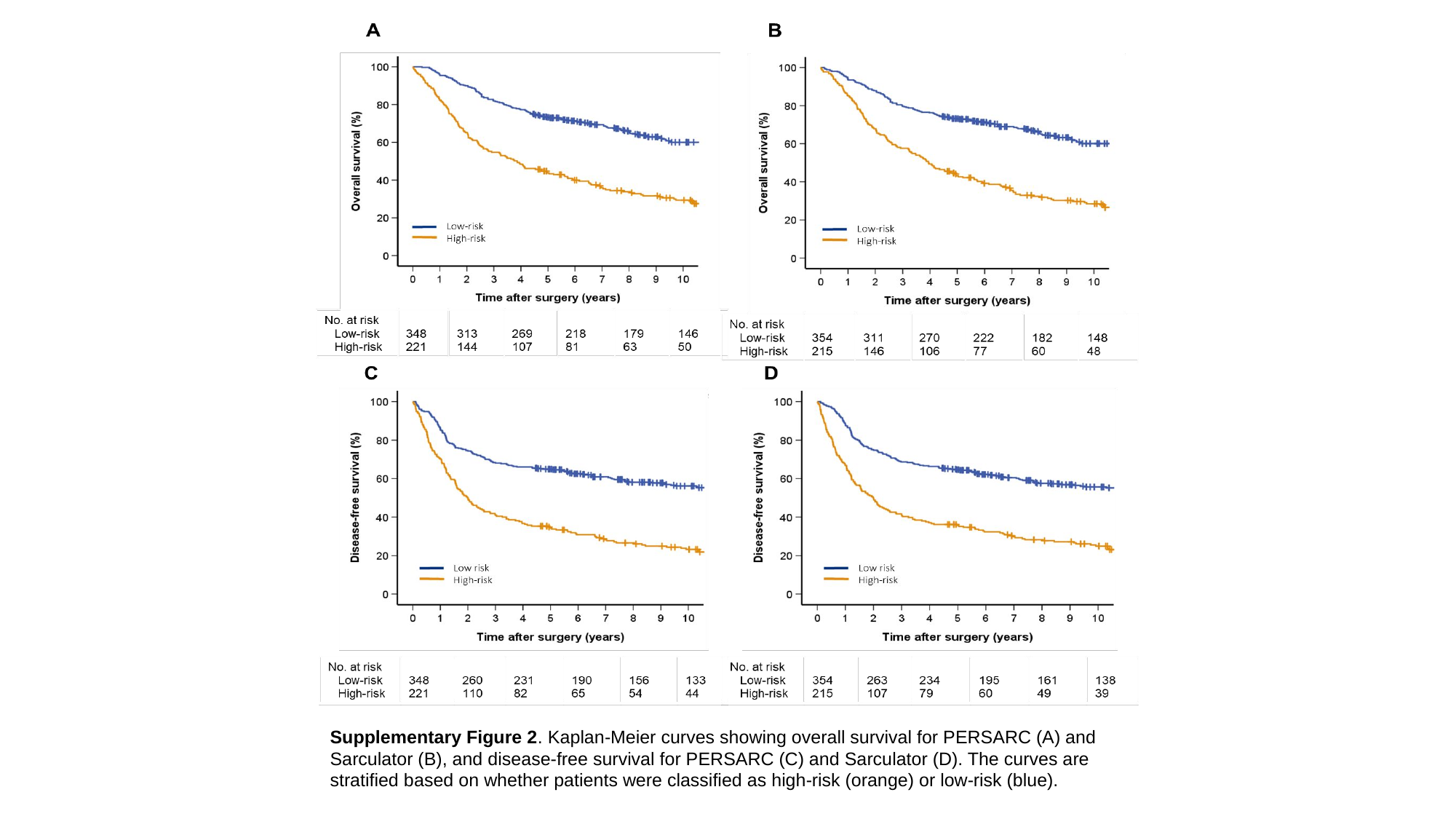

Supplementary Figure 2. Kaplan-Meier curves showing overall survival for PERSARC (A) and Sarculator (B), and disease-free survival for PERSARC (C) and Sarculator (D). The curves are stratified based on whether patients were classified as high-risk (orange) or low-risk (blue).

## Slide 3
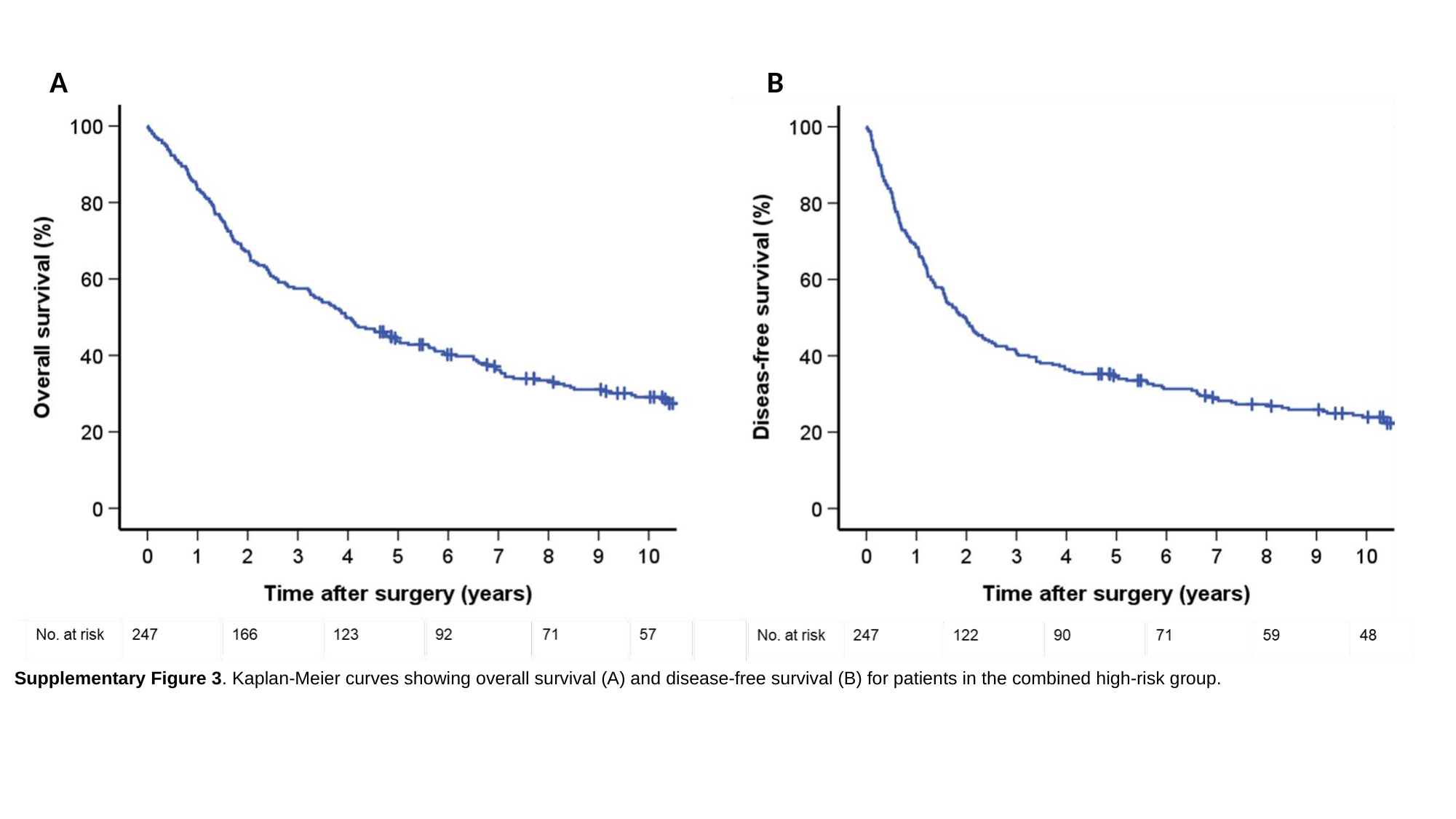

A
B
Supplementary Figure 3. Kaplan-Meier curves showing overall survival (A) and disease-free survival (B) for patients in the combined high-risk group.

## Slide 4
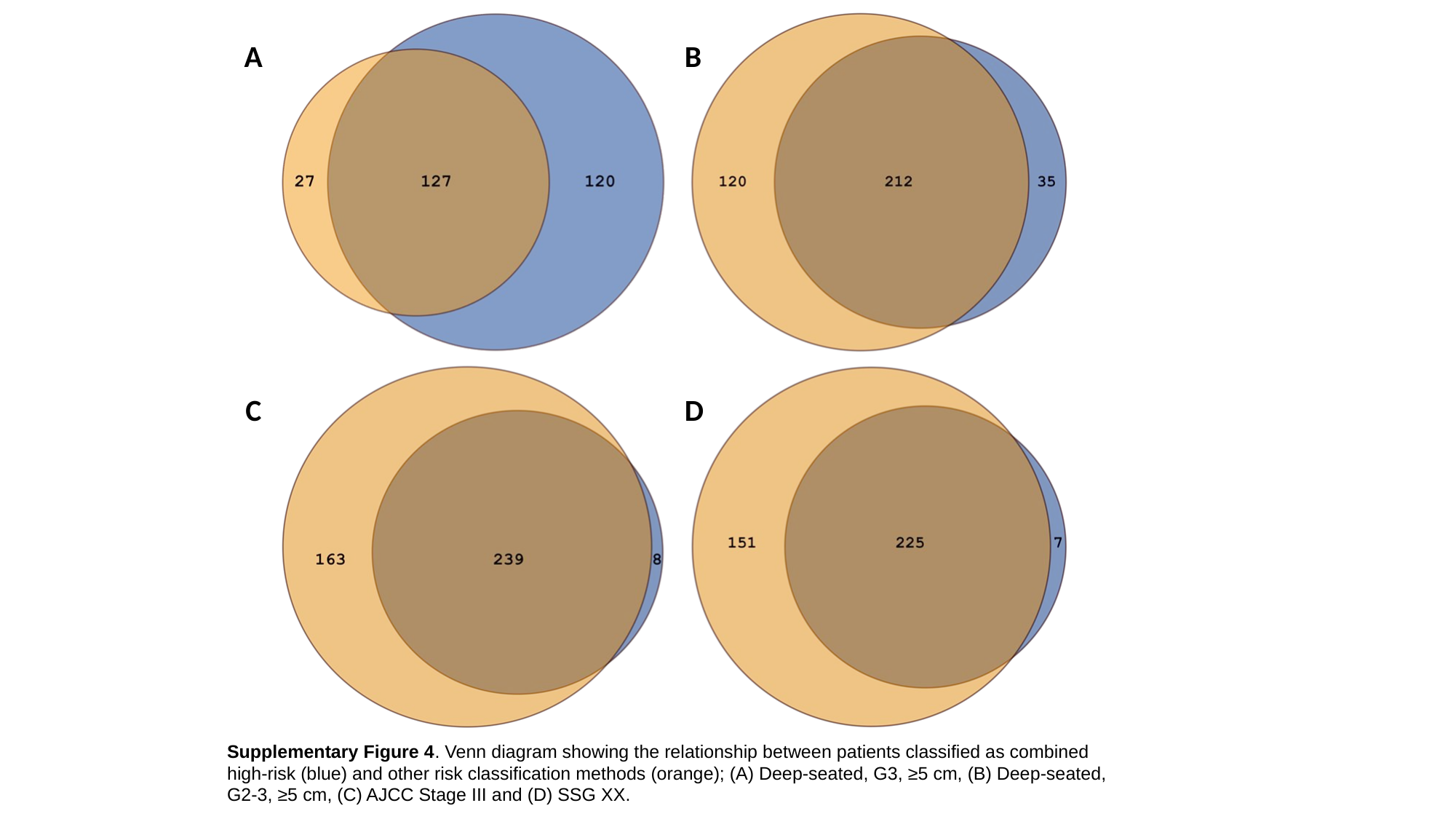

A
B
C
D
Supplementary Figure 4. Venn diagram showing the relationship between patients classified as combined high-risk (blue) and other risk classification methods (orange); (A) Deep-seated, G3, ≥5 cm, (B) Deep-seated, G2-3, ≥5 cm, (C) AJCC Stage III and (D) SSG XX.

## Slide 5
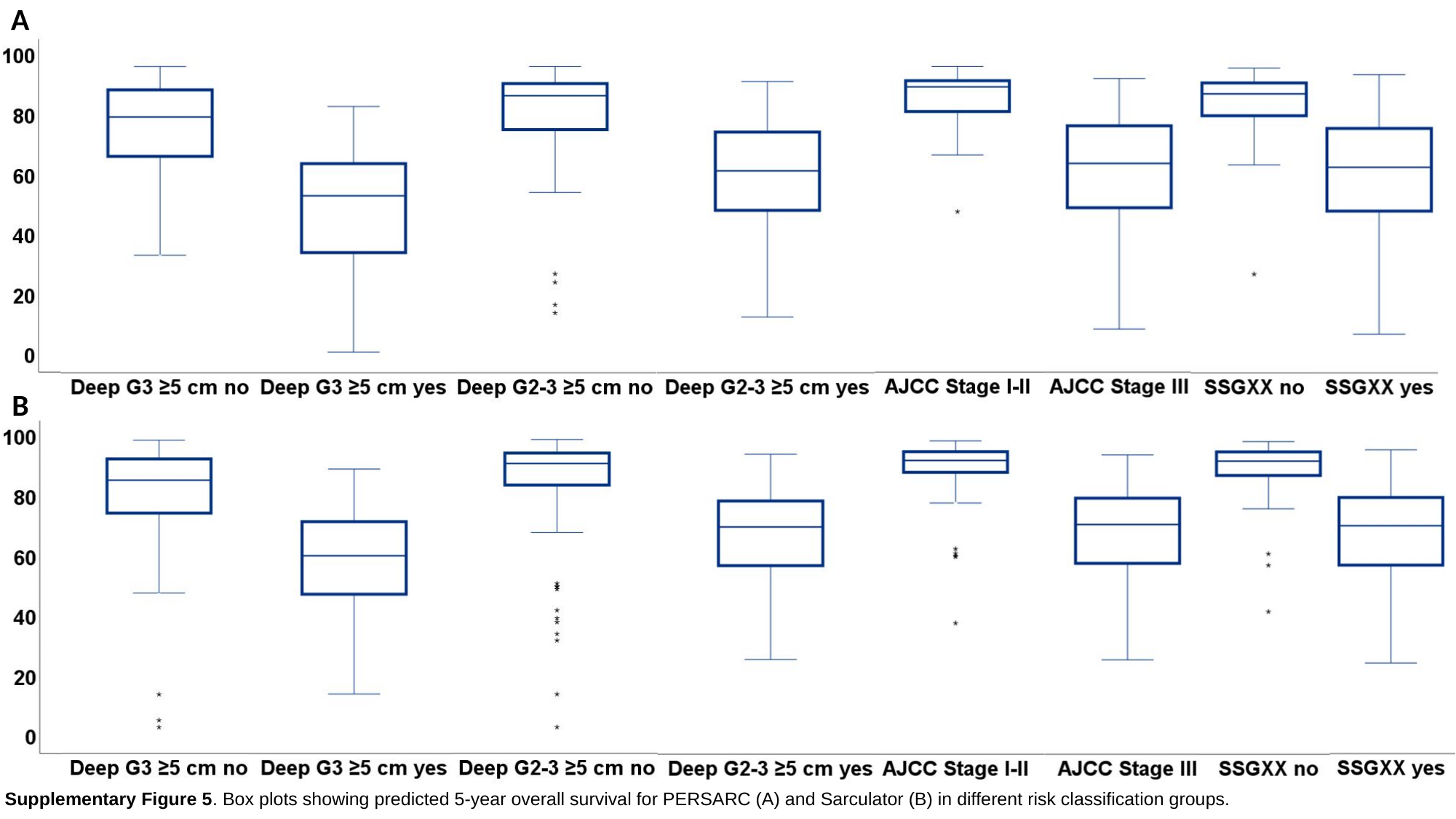

A
B
Supplementary Figure 5. Box plots showing predicted 5-year overall survival for PERSARC (A) and Sarculator (B) in different risk classification groups.
