# Supplementary material for: A comparison of the risk prediction models PERSARC and Sarculator in patients with localized soft tissue sarcoma of the extremities and trunk wall
Source: ESMO Open. 2025 Jul 24;10(8):105517. doi: 10.1016/j.esmoop.2025.105517 (PMC12312030; doi:10.1016/j.esmoop.2025.105517)
Supplement: Supplementary Tables [file mmc2.docx]

|  | PERSARC  n=32 | Sarculator  n=26 | Total  n=58 |
| --- | --- | --- | --- |
| Age at surgery, median (range) | 75 (19-92) | 60 (23-76) | 66 (19-92) |
| Sex |  |  |  |
| Female | 10 (31.3) | 7 (26.9) | 17 (29.3) |
| Male | 22 (68.8) | 19 (73.1) | 41 (70.7) |
| Primary tumor location |  |  |  |
| Lower extremity | 19 (59.4) | 13 (50.0) | 32 (55.2) |
| Upper extremity | 4 (12.5) | 5 (19.2) | 9 (15.5) |
| Trunk wall | 9 (28.1) | 8 (30.8) | 17 (29.3) |
| Histological subtype |  |  |  |
| Myxofibrosarcoma | 9 (28.1) | 4 (15.4) | 13 (22.4) |
| UPS | 14 (43.8) | 0 (0) | 14 (24.1) |
| Leiomyosarcoma | 1 (3.1) | 5 (19.2) | 6 (10.3) |
| Synovial sarcoma | 0 (0) | 4 (15.4) | 4 (6.8) |
| Liposarcomaᵃ | 3 (9.4) | 4 (15.4) | 7 (12.1) |
| MPNST | 1 (3.1) | 0 (0) | 1 (1.7) |
| Extraskeletal myxoid chondrosarcoma | 1 (3.1) | 1 (3.8) | 2 (3.4) |
| Angiosarcoma | 0 (0) | 5 (19.2) | 5 (8.6) |
| Not classified | 1 (3.1) | 2 (7.7) | 3 (5.2) |
| Otherᵇ | 2 (6.3) | 1 (3.8) | 3 (5.2) |
| Tumor size (cm), median (range) | 8.1 (3.7-16.0) | 9.4 (1.7-25.0) | 8.8 (1.7-25.0) |
| Tumor depth |  |  |  |
| Subcutaneous | 5 (15.6) | 5 (19.2) | 10 (17.2) |
| Deep | 27 (84.4) | 21 (80.0) | 48 (82.8) |
| Malignancy grade |  |  |  |
| FNCLCC 2 | 17 (53.1) | 12 (46.2) | 29 (50) |
| FNCLCC 3 | 15 (46.9) | 14 (53.8) | 29 (50) |
| Surgical margin |  |  |  |
| R0 | 24 (75.0) | 25 (96.2) | 49 (84.5) |
| R1 | 8 (25.0) | 1 (3.8) | 9 (15.5) |
| Neoadjuvant/adjuvant chemotherapy |  |  |  |
| Yes | 6 (18.8) | 14 (53.8) | 20 (34.5) |
| No | 26 (81.3) | 12 (46.2) | 38 (65.5) |
| Neoadjuvant/adjuvant radiotherapy |  |  |  |
| Yes | 9 (28.1) | 23 (88.5) | 32 (55.2) |
| No | 23 (71.9) | 3 (11.5) | 26 (44.8) |
| Distant metastasis |  |  |  |
| Yes | 16 (50) | 18 (69.2) | 34 (58.6) |
| No | 16 (50) | 8 (30.8) | 24 (41.4) |
| Local recurrence |  |  |  |
| Yes | 6 (18.8) | 5 (19.2) | 11 (19.0) |
| No | 26 (81.3) | 21 (80.8) | 47 (81.0) |

**Supplementary Table S1**. Demographic, clinical and pathological characteristics of patients classified as high-risk by one model.

Data are number of patients (%) unless otherwise specified.

ᵃPleomorphic or dedifferentiated; ᵇOther subtypes included extraskeletal osteosarcoma (2) and epithelioid sarcoma (1).

UPS, undifferentiated pleomorphic sarcoma; MPNST, malignant peripheral nerve sheath tumor; FNCLCC, Fédération Nationale des Centers de Lutte Contre Le Cancer; R0, complete resection with microscopically negative margins; R1, complete resection with microscopically positive margins.

**Supplementary Table S2** Outcome data for PERSARC and Sarculator high-risk groups and the total cohort.

|  | **PERSARC high-risk (n=221)** | **Sarculator high-risk (n=215)** | **Combined high-risk (n=247)** | **Total cohort**  **(n=664)** |
| --- | --- | --- | --- | --- |
| **Disease recurrenceᵃ**  Distant metastasis  Local recurrence  Distant and local recurrence  No recurrence | 72 (32.6)  13 (5.9)  24 (10.9)  112 (50.7) | 73 (34.0)  12 (5.6)  25 (11.6)  105 (48.8) | 84 (34.0)  13 (5.3)  30 (12.1)  120 (48.6) | 162 (24.4)  24 (3.6)  48 (7.2)  430 (64.8) |
| **Site of distant metastasis^a,b^**  Lung/pleura  Bone  Soft tissue or subcutaneous  Lymph node  Brain  Peritoneal or retroperitoneal | 80 (36.2)  3 (1.4)  9 (4.1)  3 (1.4)  1 (0.5) | 80 (37.2)  5 (2.3)  7 (3.2)  3 (1.4)  2 (0.9)  1 (0.5) | 94 (38.1)  5 (2.0)  9 (3.6)  3 (1.2)  2 (0.8)  1 (0.4) | 162 (24.4)  16 (2.4)  22 (3.4)  6 (0.9)  2 (0.3)  2 (0.4) |
| **Survival status^c^**  Dead  Alive | 169 (76.5)  52 (23.5) | 163 (75.8)  52 (24.2) | 189 (76.5)  58 (23.5) | 349 (52.6)  315 (47.4) |
| **Overall survival^c^**  Median  5-year  10-year | 46 months  44 %  29 % | 47 months  43 %  28 % | 47 months  44 %  29 % | 144 months  67 %  53 % |
| **Disease-free survival^c^**  Median  5-year  10-year | 24 months  36 %  25 % | 24 months  34 %  23 % | 23 months  34 %  24 % | 70 months  58 %  47 % |

ᵃData are number of patients (%); ᵇLocation of first distant metastasis; **^c^**Median survival is given in months, 5-year and 10-year estimated survival is presented as percentages.

|  | **PERSARC n=221** | **Sarculator n=215** | **Combined n=247** |
| --- | --- | --- | --- |
| Number of patients (%) | | | |
| Age at surgery, median (range) | 72 (19-94) | 71 (23-94) | 70 (19-94) |
| Sex  Female  Male | 104 (47.1)  117 (49.3) | 101 (47.0)  114 (53.0) | 111 (44.9)  136 (55.1) |
| Primary tumor location  Lower extremity  Upper extremity  Trunk wall | 145 (65.6)  27 (12.2)  49 (22.2) | 139 (64.7)  28 (13.0)  48 (22.3) | 158 (64.0)  32 (13.0)  57 (23.1) |
| Histological subtype  Myxofibrosarcoma  UPS  Leiomyosarcoma  Liposarcomaᵃ  Synovial sarcoma  MPNST  Extraskeletal myxoid chondrosarcoma  Angiosarcoma  Not classified  Otherᵇ | 93 (42.1)  49 (22.2)  18 (8.1)  24 (10.9)  5 (2.3)  5 (2.3)  2 (0.9)  5 (2.3)  7 (3.2)  13 (5.9) | 88 (40.9)  35 (16.3)  22 (10.2)  25 (11.6)  9 (4.2)  4 (1.9)  2 (0.9)  10 (4.7)  8 (3.7)  12 (5.6) | 97 (39.3)  49 (19.8)  23 (9.3)  28 (9.3)  9 (3.6)  5 (2.0)  3 (1.2)  10 (4.0)  9 (3.6)  14 (5.7) |
| Tumor size (cm) median (range) | 12.7 (3.2-40.0) | 13 (1.7-40.0) | 12.5 (1.7-40) |
| Tumor depth  Subcutaneous  Deep | 28 (12.7)  193 (87.3) | 28 (13.0)  187 (87.0) | 33 (13.4)  214 (86.6) |
| Malignancy grade  FNCLCC 2  FNCLCC 3 | 77 (34.8)  144 (65.2) | 72 (33.5)  143 (66.5) | 89 (36.0)  158 (64.0) |
| Surgical margin  R0  R1 | 177 (80.1)  44 (19.9) | 178 (82.8)  37 (17.2) | 202 (81.8)  45 (18.2) |
| Neoadjuvant/adjuvant chemotherapy  Yes  No | 50 (22.6)  171 (77.4) | 58 (27.0)  157 (73.0) | 64 (25.9)  183 (74.1) |
| Neoadjuvant/adjuvant radiotherapy  Yes  No | 108 (48.9)  113 (51.5) | 122 (56.7)  93 (43.3) | 131 (53.0)  116 (47.0) |

**Supplementary Table S3**. Baseline characteristics and treatment for patients in PERSARC, Sarculator and combined high-risk groups.

ᵃPleomorphic or dedifferentiated liposarcoma; ᵇOther subtypes include extraskeletal osteosarcoma, epithelioid sarcoma, fibrosarcoma, sclerosing epithelioid fibrosarcoma, pleomorphic rhabdomyosarcoma and sclerosing rhabdomyosarcoma.

UPS, undifferentiated pleomorphic sarcoma; MPNST, malignant peripheral nerve sheath tumor; FNCLCC, Fédération Nationale des Centers de Lutte Contre Le Cancer; R0, complete resection with microscopically negative margins; R1, complete resection with microscopically positive margins.
